# Aqueous-Based Binary Sulfide Nanoparticle Inks for Cu_2_ZnSnS_4_ Thin Films Stabilized with Tin(IV) Chalcogenide Complexes

**DOI:** 10.3390/nano9101382

**Published:** 2019-09-26

**Authors:** Han Wang, Amrita Yasin, Nathaniel J. Quitoriano, George P. Demopoulos

**Affiliations:** Materials Engineering, McGill University, Montreal, QC H3A 0C5, Canada; han.wang5@mail.mcgill.ca (H.W.); amrita.yasin@mail.mcgill.ca (A.Y.); nate.quitoriano@mcgill.ca (N.J.Q.)

**Keywords:** Cu_2_ZnSnS_4_, kesterite, photovoltaics, semiconductor films, nanoparticles, spin coating, aqueous ink formulation

## Abstract

Cu_2_ZnSnS_4_ (CZTS) is a promising semiconductor material for photovoltaic applications, with excellent optical and electronic properties while boasting a nontoxic, inexpensive, and abundant elemental composition. Previous high-quality CZTS thin films often required either vacuum-based deposition processes or the use of organic ligands/solvents for ink formulation, which are associated with various issues regarding performance or economic feasibility. To address these issues, an alternative method for depositing CZTS thin films using an aqueous-based nanoparticle suspension is demonstrated in this work. Nanoparticles of constituent binary sulfides (Cu_x_S and ZnS) are stabilized in an ink using tin(IV)-based, metal chalcogenide complexes such as [Sn_2_S_6_]^4−^. This research paper provides a systematic study of the nanoparticle synthesis and ink formulation via the enabling role of the tin chalcogenide complexing power, the deposition of high-quality CZTS thin films via spin coating and annealing under sulfur vapor atmosphere, their structural characterization in terms of nanocrystal phase, morphology, microstructure, and densification, and their resultant optoelectronic properties.

## 1. Introduction

Cu_2_ZnSnS_4_ (CZTS) in the kesterite crystal structure [1] is a promising absorber layer material for next-generation thin film solar cells, featuring desirable optoelectronic properties and a composition made of earth-abundant and nontoxic elements, the latter in contrast to current commercialized thin film photovoltaics such as CuIn_1−x_ Ga_x_Se_2_ (CIGS) and CdTe [2,3,4]. While CZTS thin films have been successfully deposited using vacuum-based processing methods such as sputtering [5,6] and thermal evaporation [7,8], there is much interest in nonvacuum processing techniques, as vacuum-based methods are generally very costly [9]. A variety of nonvacuum-based processing methods have been used to successfully deposit high-quality CZTS thin films, including nanocrystal dispersions [10], solution processing [11], spray pyrolysis [12], and electrodeposition [13]. The current record efficiency for CZTS-based photovoltaics standing at 12.6% was obtained using a hydrazine-based solution via spin coating followed by annealing in a selenium atmosphere [14]. Selenium is often used in CZTS devices for its ability to tune the bandgap of CZTS between 1.0 eV and 1.5 eV via the partial replacement of sulfur in the crystal structure, forming Cu_2_ZnSn(S,Se)_4_ [15]. The introduction of selenium is also beneficial to the film morphology, as its presence in the annealing of CZT(S,Se) films provides a reactive sintering environment which densifies the film and enhances grain size and compositional homogeneity, which are favorable for photovoltaic performance [16,17,18]. The benefits of selenization must be balanced against the hazardous toxic nature of selenium vapors. The record efficiency for a non-selenized CZTS photovoltaic device is 11% [19].

While nonvacuum based deposition techniques are attractive options because they generally offer facile and scalable deposition of CZTS absorber layers with high throughput and low processing cost, many of the techniques employ toxic, organic solvents, especially in the case of colloidal nanocrystals that are often made using hot injection procedures [20]. These organics are difficult to fully remove from the absorber film and leave a carbonaceous residue or develop a carbon-rich, fine grain layer that can hamper photovoltaic performance [21,22,23]. Thus, it would be beneficial to use an inorganic ligand to stabilize CZTS nanocrystal inks and avoid residual carbon contamination during annealing of the film [24]. Hydrazine is a carbon-free solvent which readily enables the formation of metal chalcogenide complexes that act as molecular, inorganic ligands, but it is highly volatile and dangerous, owing to both its toxicity and explosive nature, making it therefore difficult to justify as a feasible deposition method [25]. However, an aqueous ammonium sulfide solution can be used instead of hydrazine solvent to form metal chalcogenide complexes, hence offering a safer and more environmentally friendly alternative [25,26,27].

Thiostannate(IV) complexes (e**.**g.**,** [Sn_2_S_6_]**^4−^**) dissolved in aqueous ammonium sulfide can act as inorganic ligands with strong affinity for nanocrystal surfaces leading to colloidal stabilization [24]; in addition, these complexes can service as a source of tin, a necessary elemental component of CZTS. Zhong et al. were the first to successfully demonstrate the use of such tin chalcogenide complexes to prepare nanoinks for CZTS solar cell fabrication [28]. In their work, however, they prepared the tin chalcogenide complex by dissolving metallic tin and elemental sulfur in ammonium sulfide solution, a rather inefficient method prone to poor reproducibility due to oxidation of metallic tin without the use of a glovebox. Ritchie et al. showed that the redox process to form the tin chalcogenide complexes had poor stability, which may complicate larger scale applications [29]. Instead, a dissociative process can be used to make the tin chalcogenide complex as per the method of Krebs involving dissolution of tin(IV) sulfide into aqueous ammonium sulfide solution [25] adopted for CZTS film fabrication by Ritchie et al. [29]. In both of these CZTS aqueous nanoink works [28,29], thiourea was used as a supplementary complexing-stabilizing agent [30,31,32] for the soluble copper and zinc salts. Given that thiourea is classified as a likely human carcinogen [33], cleaner processing routes must be pursued.

An alternative method to synthesize CZTS is to introduce copper and zinc as binary sulfide nanoparticles (e.g., Cu_x_S, ZnS) instead of soluble salts that the “one-pot” methods utilize [28,29], which can be blended with SnS_2_ nanoparticles and/or [Sn_2_S_6_]^4−^ complex-bearing ammonium sulfide solution, offering a facile method of adjusting the elemental composition of CZTS, a critical parameter of its photovoltaic performance [34,35]. This method of synthesis also removes the need to use the toxic thiourea complexing agent. Another attractive feature of using binary sulfide nanocrystals instead of CZTS nanocrystals is that it can enable a reactive sintering environment without reliance on selenium during the annealing process [36]. Reactive sintering helps to achieve uniform elemental distribution, which reduces the likelihood of forming localized, detrimental secondary phases and grows larger grains which lower the impact of charge carrier trapping and recombination at interfacial features.

In this work, we utilize the thiostannate (IV) complexing power to stabilize an aqueous-based nanoink composed of nanoparticles of the constituent binary sulfides of CZTS. Copper sulfide (Cu_x_S) and zinc sulfide (ZnS) nanocrystals are synthesized in our process using a hydrothermal route, which is an inexpensive, environmentally friendly, and scalable technique for producing various nanocrystals, including metal chalcogenides [37]. Tin sulfide (SnS_2_) is separately synthesized with an ambient aqueous chemical route to prevent the hydrolysis of Sn^4+^ in water, which can form tin oxide impurities [38]. This work demonstrates the robust nature of binary sulfide nanocrystal conversion to kesterite CZTS crystalline films using an aqueous thiostannate(IV)-stabilized nanoink method with minimal secondary phases and impurities. The binary nanoparticle mixing with the thiostannate(IV) and aqueous ammonium sulfide solution allows the formulation of highly concentrated inks compared to in situ formation of nanoparticles from thiourea-metal solutions in the one-pot route [28,29], achieving a 5-10 times higher yield. The concentration of solid weight fraction of suspended nanoparticles and dilution of ammonium sulfide in water are further modulated in order to enhance the stability of the nanoink and consequently the film microstructure, morphology and density. Optical properties are characterized, showing good absorption coefficient and bandgap for CZTS films prepared using this technique.

## 2. Materials and Methods

### 2.1. Nanocrystal Synthesis

In a typical synthesis, 23 mmol copper (II) chloride dihydrate (CuCl_2_·2H_2_O) and 12 mmol zinc chloride (ZnCl_2_) were added to 20 mL deionized water and magnetically stirred for several minutes until fully dissolved to form the cationic solution. In a separate flask, 47 mmol of sodium sulfide nonahydrate (Na_2_S·9H_2_O) was dissolved into 40 mL of deionized water to form the anionic solution. A peristaltic pump was used to transfer the cationic solution at a rate of 2 mL/minute into the anionic solution while under constant magnetic stirring at 800 RPM. After additional stirring for 30 min, the precursor mixture was heated at 180 °C for 24 h using a Parr pressure vessel (125 mL capacity) with a polytetrafluoroethylene liner. The nanoparticle mixture was cooled naturally to room temperature and washed using H_2_O and ethanol by centrifugation, then dried under vacuum at 60 °C overnight.

The synthesis for SnS_2_ was adapted from literature [25], combining sodium stannate trihydrate (Na_2_SnO_3_·3H_2_O) and an excess of Na_2_S·9H_2_O into boiling deionized water, followed by the slow addition of 1 M hydrochloric acid until SnS_2_ crystals precipitated at a solution pH of 4. Crystals were then collected and washed via centrifugation using water and acetone and dried in air.

### 2.2. Ink Deposition and Sulfurization

For a typical ink, 4 mmol of the Cu_x_S/ZnS mixture and 2 mmol of SnS_2_ were mixed into a 1:9 dilution of 0.5 mL aqueous ammonium sulfide ((NH_4_)_2_S) **(**40–48 wt % in H_2_O) in 4.5 mL of deionized water. Inks were briefly ultrasonicated and then magnetically stirred for a few hours until a homogeneous dispersion was obtained. The inks were deposited onto 1 square inch soda-lime glass substrates by spin coating with a Laurell WS-650 spin coater at 3000 RPM for 30 s and heated on a hot plate in air at 300 °C for 5 min. This spin coating procedure was repeated five times to achieve the desired thickness for the absorber layer. A final sulfurization step was used to convert the binary sulfides into large kesterite grains, using an MTI OTF-1200X-RTP furnace, purged with Argon gas three times. Films were placed into a graphite crucible with elemental sulfur equivalent to 0.15 atm vapor pressure and annealed for 30 min at 550 °C with a ramp rate of 2 °C/second.

### 2.3. Materials Characterization

Phase characterization was performed on powder and thin films samples using X-ray diffraction (XRD; Bruker, Madison, WI, USA) with a Cu Kα source (λ = 1.54056 Å) and VANTEC area detector. The instrument was operated at 40 kV and 40 mA. Additional phase data for thin films and photoluminescence (PL) data was gathered with an inVia confocal Raman microscope (Renishaw, Wotton-under-Edge, UK) with a laser wavelength of 514 nm. X-ray photospectroscopy (XPS; Thermo Fisher Scientific, Waltham, MA, USA) analysis was performed to determine the chemical oxidation states of the CZTS elements and for impurity level analysis. A flood gun was used to prevent charging on the sample, and the spectra were shifted for charge correction to the C1s peak, and spectra were captured after ion etching of adsorbed carbon contamination. Scanning electron microscopy (SEM; Hitachi SU3500, Tokyo, Japan) was used to take images of the film morphology with an accelerating voltage of 20 kV. This was used in conjunction with an energy-dispersive x-ray spectroscopy system (EDS; Oxford, Abingdon, UK) for additional composition information in relation to the film microstructure. Cross-section images of thin films were taken using a different microscope (Hitachi SU8000, Tokyo, Japan) at an accelerating voltage of 2 kV. Optical properties including transmittance and reflectance spectra were gathered with a QEX10 system (PV Measurements, Point Roberts, WA, USA).

## 3. Results & Discussion

### 3.1. Binary Sulfide Characterization and Conversion to Kesterite Phase

**Figure 1:**
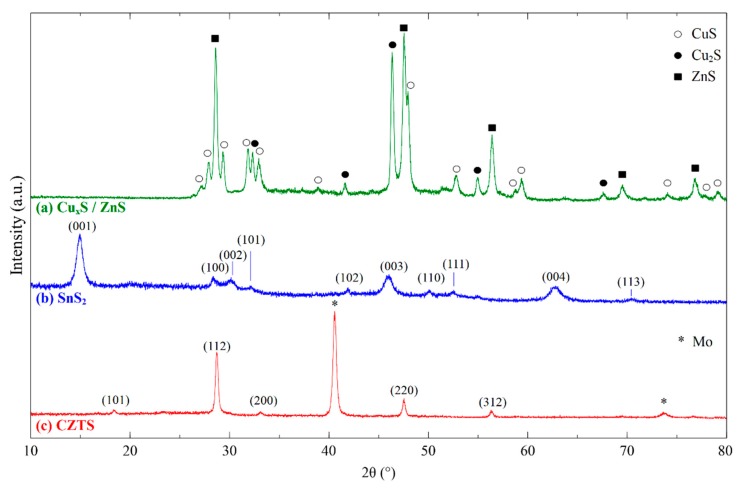
(**a**) XRD patterns of Cu_x_S, ZnS nanocrystals synthesized from hydrothermal treatment. Reference peaks correspond to sphalerite ZnS, covellite CuS, and digenite Cu_2_S. (**b**) SnS_2_ thin film XRD pattern taken from a decomposed thiostannate (IV) complex, matched to berndtite SnS_2_. (**c**) CZTS thin film XRD pattern deposited on a Mo-coated soda lime glass substrate, with peaks matched to kesterite Cu_2_ZnSnS_4_ and sputtered Mo of the substrate.

Figure 1a shows an XRD pattern of nanocrystals synthesized after hydrothermal synthesis, corresponding to a combination of ZnS and Cu_x_S. Sphalerite zinc sulfide (PDF No. 05-0566) is a stable structure under these conditions, but the copper sulfide showed a mixed phase of covellite (PDF No. 06-0464) and digenite (PDF No. 02-1284), despite the excess presence of sulfur. The XRD of a thin film of SnS_2_, deposited in a similar manner as described in the methodology for CZTS, is shown in Figure 1b, in order to verify the crystallization of SnS_2_ after being dissolved and forming thiostannate(IV) complex in aqueous ammonium sulfide [27]. Peaks were matched to berndtite (PDF No. 23-0677), and no impurity phases were detected.

**Figure 2:**
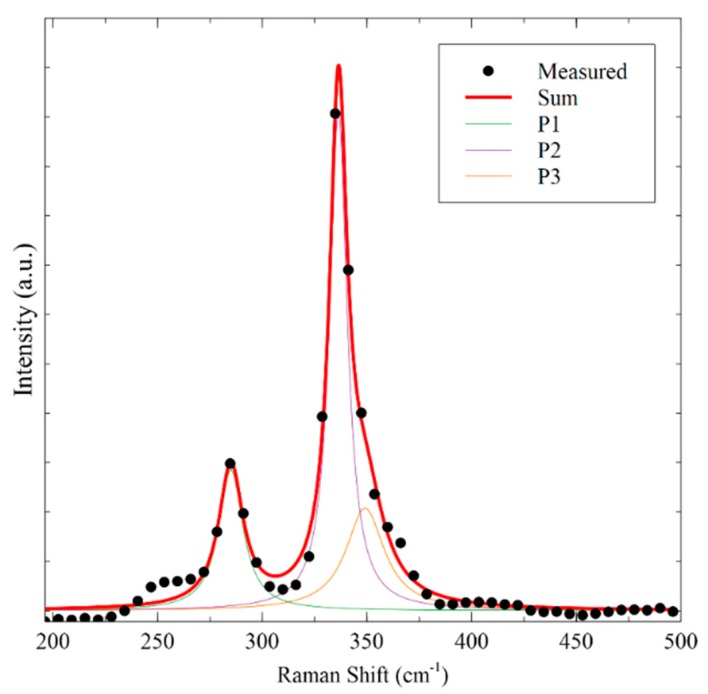
Raman spectrum for CZTS thin film, fitted using Lorentzian curves. Peaks were determined to be at 285 cm^−1^ (P1), 336 cm^−1^ (P2), and 349 cm^−1^ (P3).

After sulfurization of the binary sulfides, a phase transition was observed to the crystallized CZTS phase. Figure 1c shows the XRD diffraction patterns for a CZTS thin film deposited from the binary sulfide nanoink after sulfurization at 550 °C. Diffraction peaks were found at 18.35°, 28.72°, 33.13°, 47.54°, and 56.35°, which correspond to (101), (112), (200), (220), and (312) planes of the kesterite CZTS crystal structure (PDF No. 26-0575), respectively, which is the most thermodynamically stable and expected phase of CZTS despite energetic similarities in the crystal structure to stannite [39,40]. Lattice parameter calculations were made for the XRD patterns which also support the presence of the kesterite phase, found in Table S1 [41]. Binary sulfide phases for Cu_x_S or SnS_2_ from the ink were not detected, and no additional secondary or impurity phases were seen. However, XRD is not a conclusive phase analysis technique for the CZTS system due to the potential presence of overlapping constituent phase peaks such as ZnS and Cu_2_SnS_3_ [42], so Raman spectroscopy is used in conjunction to confirm the exclusive presence of kesterite CZTS phase. In Figure 2, the Raman spectrum is shown for the CZTS film with peaks detected at 336 cm^−1^ and 285 cm^−1^, and a shoulder peak found at 349 cm^−1^. The dominant peak at 336 cm^−1^ is associated with the A1 mode, originating from the vibration of sulfur atoms in the lattice [43]. These values correspond to CZTS peaks found in the literature, and no notable impurity peaks from the binary sulfides such as Cu_2−x_S or SnS_2_ are seen in the spectrum. The shoulder at 349 cm^−1^ is difficult to distinguish at the measured resolution whether it correlates with sphalerite ZnS or kesterite CZTS [44]. However, the A1 mode observed is shifted lower from the expected values at 337–339 cm^−1^, which may indicate a disordered kesterite structure, which primarily arises from Cu and Zn antisite defects [45].

**Figure 3:**
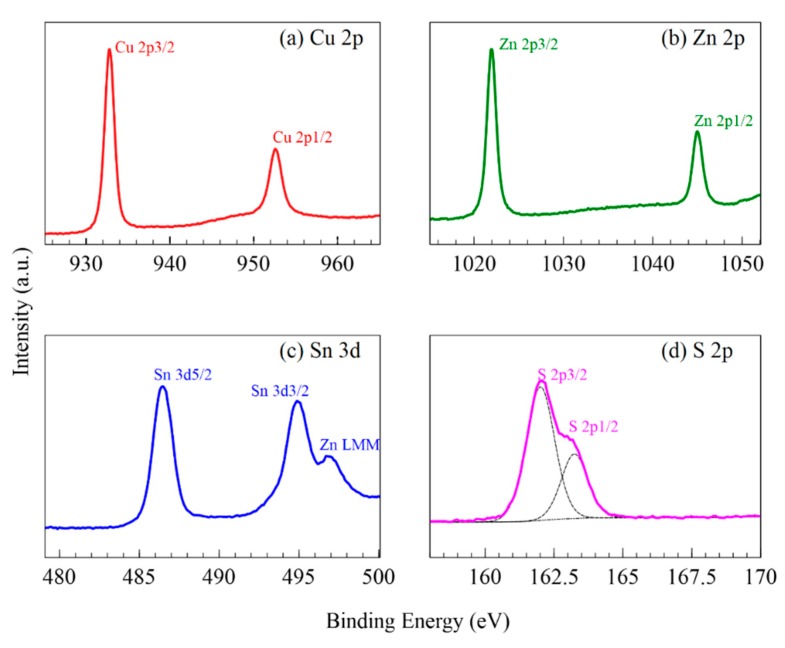
XPS high resolution spectra of CZTS thin films of (**a**) Cu 2p, (**b**) Zn 2p, (**c**) Sn 3d, and (**d**) S 2p.

The XPS profiles for Cu 2p, Zn 2p, Sn 3d, and S 2p are shown in Figure 3, which give insight to the oxidation state of the elements in the CZTS thin film. Cu 2p features peaks at 952.6 eV for Cu 2p1/2 and 932.8 eV for Cu 2p3/2 with a peak splitting value of 19.8 eV, which is characteristic of Cu^+^. No additional satellite peaks detected in the spectra that represent Cu^2+^, showing a reductive ability of the annealing process for the mixed copper sulfide phases in the hydrothermal product [46]. The Zn 2p spectra indicate the presence of Zn^2+^ with peaks at 1045.0 eV and 1022.0 eV which correspond to Zn 2p1/2 and Zn 2p3/2 respectively and a peak energy difference of 23.0 eV. The Sn 3d profile contains peaks at 494.9 eV and 486.5 eV for Sn 3d3/2 and Sn 3d5/2 and a peak separation of 8.4 eV, which indicate the presence of Sn^4+^. There was an additional shoulder peak at 497.0 eV, which is related to a Zn LMM Auger line that has been observed in some instances for CZTS [47,48]. Upon deconvolution, peaks were found at 163.2 eV and 162.0 eV for S 2p3/2 and S 2p1/2, with a peak separation of 1.2 eV. This is consistent with sulfur in the S^2−^ state, and no extraneous peaks for sulfite or sulfate were detected [48]. The oxidation states for Cu, Zn, Sn, and S found with XPS analysis correspond to the correct oxidation states of the CZTS crystal structure [46,47,48,49].

### 3.2. Optical Properties of CZTS Films

**Figure 4:**
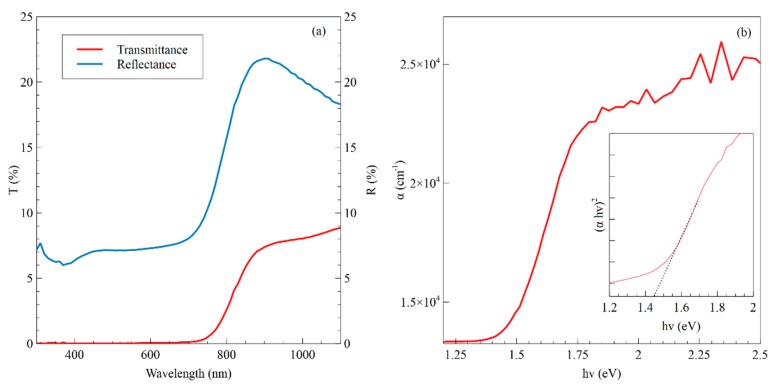
(**a**) Transmittance and reflectance measurements for a typical CZTS thin film using the binary nanoink. (**b**) Absorption coefficient calculated for CZTS film, derived from transmittance and reflectance. The inset displays a Tauc plot based on absorption coefficient, with linear portion of plot fit extrapolated for the optical bandgap at 1.44 eV.

Transmittance and reflectance measurements were conducted to confirm the optical properties for the CZTS films and to determine the optical bandgap, which are displayed in Figure 4a. The films showed high absorbance in the visible spectrum up to 750 nm, with negligible transmittance and an average reflectance of about 7%. There was a substantial increase in both parameters upon entering the infrared regime, where an absorption edge is detected. The absorption coefficient was derived using the transmittance and reflectance data and shown in Figure 4b, with values above 10^4^ cm^−1^ that is on par with values found in literature [50]. The inset graph in Figure 4b shows a Tauc plot constructed for CZTS comparing *hν* to (α*hν*)^1/n^, where n = 1/2 for a direct bandgap semiconductor. The optical bandgap is determined using extrapolation of the linear regime to be 1.44 eV, which is in agreement with reported values for the theoretical and measured bandgap of CZTS [51,52,53].

**Figure 5:**
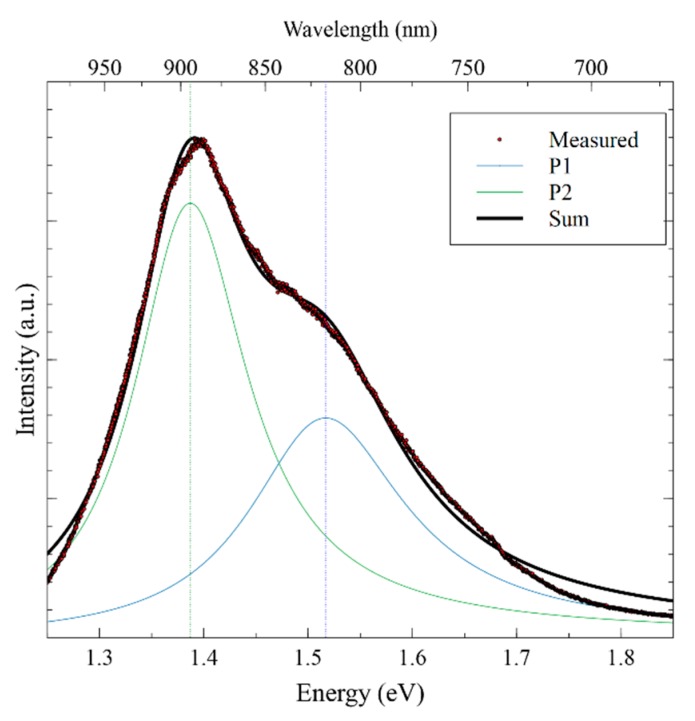
Photoluminescence and fitted peaks for CZTS thin film taken at room temperature.

Room-temperature photoluminescence (PL) was performed to detect photoexcitation characteristics in the absorber layer, which is shown in Figure 5. There is a broad photoluminescence signal with two convoluted bands seen at 1.38 eV and 1.51 eV. The 1.51 eV PL band can be attributed to band-to-band recombination mechanism, which aligns with the theoretical bandgap of polycrystalline CZTS. The PL band centered at 1.38 eV is likely due to band-to-tail recombination, indicating the presence of defect states [54]. A mixture of ordered and disordered kesterite may explain the broadness of the PL bands. This is also supported by the shift detected in the Raman spectra as previously mentioned. The issue of disordered kesterite is a significant problem in many CZTS works, particularly due to the high likelihood of Cu and Zn antisite defects due to the similar atomic radii and coordination of the two elements [1,55]. The width of the PL spectrum may also be impacted by the presence of trap centers in the bulk of the film [56].

### 3.3. Improving Ink Stability and Film Morphology

**Figure 6:**
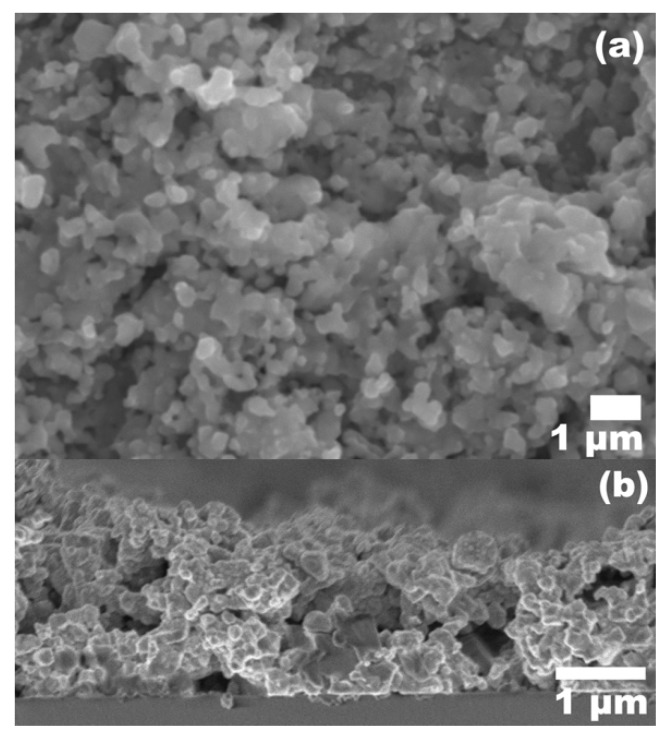
Microstructure of typical sulfurized CZTS thin film at 550 °C. (NH_4_)_2_S:H_2_O dilution was 1:9, and solid weight concentration was 170 mg/mL. (**a**) Top-down image, (**b**) Cross-section view.

The aqueous nanoink containing the binary sulfides with the tin chalcogenide complex showed success in forming the kesterite CZTS phase upon sulfurization. However, deposited films exhibited unsuitable film morphology for solar devices. Figure 6 shows SEM images taken for a typical thin film, featuring a porous microstructure with low grain growth, high surface roughness, and formation of large gaps or cracks which can extend throughout the thickness of the film, as indicated by the cross-section image in Figure 6b. Although the binary sulfides are expected to provide a reactive sintering environment [36], more analysis is necessary to establish optimal grain growth conditions for this methodology. Low coating density is detrimental to device performance and should be avoided, due to the increased presence of voids and cracks causing surface defects and a film with reduced grain size can result in unfavorable carrier transport [57].

**Figure 7:**
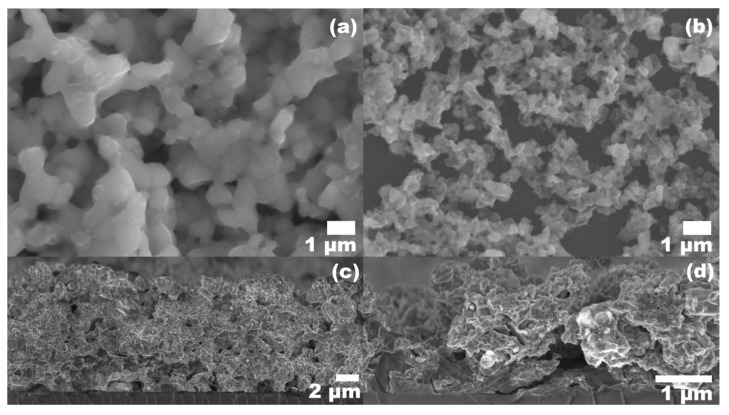
Microstructure of CZTS thin films obtained at different solid concentration at 1:9 dilution of (NH_4_)_2_S:H2O. (**a**,**c**) 550 mg/mL planar and cross-sectional view, (**b**,**d**) 100 mg/mL planar and cross-sectional view, respectively.

The solid weight fraction of the aqueous nanoinks was adjusted to improve the substrate coverage, film density, and grain size. (NH_4_)_2_S:H_2_O dilution was kept constant at 1:9. Top view and cross section morphologies of films made using inks at 550 mg/mL and 100 mg/mL can be seen in SEM images in Figure 7. With the 550 mg/mL ink, there is a noticeable improvement in the film uniformity, with enhanced sintering between the nanoparticles. The cross section in Figure 7c indicates a more densely packed microstructure with a reduction in voids within the film. The lower ink concentration of 100 mg/mL resulted in an abundance of voids and an irregular film morphology. The planar view in Figure 7b shows loose nanoparticle networks or clusters of nanoparticles that are disconnected from one another, with incomplete substrate coverage and uneven film thickness. The increased solid loading of the 550 mg/mL nanoink forms a more cohesive structure, and the grain size is notably improved. However, the thickness of the film made using the 550 mg/mL ink was relatively high when depositing the same number of layers as the other film conditions, which can be seen in the cross section in Figure 7c. A reduction in the number of layers deposited via spin coating is recommended for more concentrated inks.

**Figure 8:**
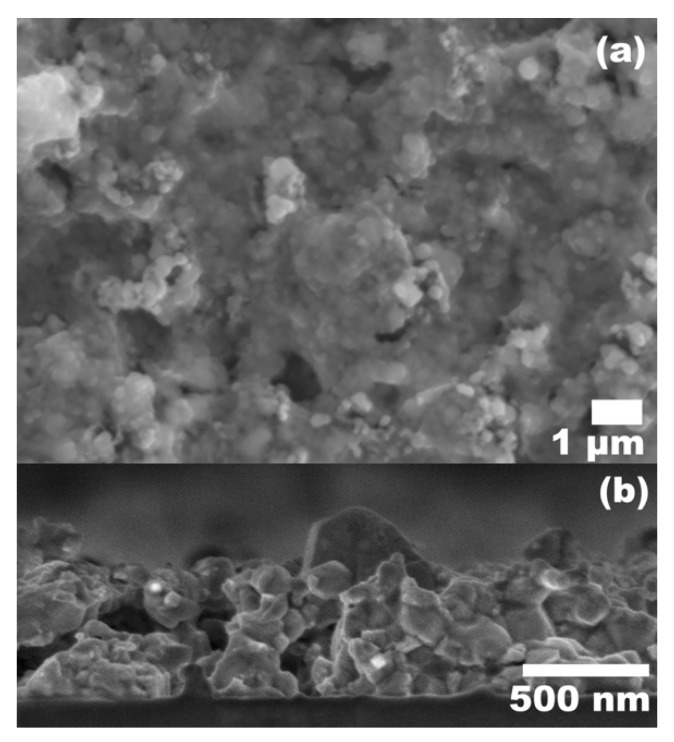
CZTS film produced using nanoink with (NH_4_)_2_S:H_2_O dilution of 1:50 and nanoparticle concentration of 170 mg/mL. (**a**) Planar view, (**b**) Cross-section view.

The (NH_4_)_2_S concentration in the solvent was adjusted to enhance the ink stability, keeping constant the nanoparticle concentration of 170 mg/mL. In the experiments which used a controlled dilution level of 1:9 relative to H_2_O, the thiostannate (IV) complex was formed by fully dissolving the SnS_2_ prior to the addition of the other binary sulfides. These inks showed instability and particles visibly sedimented over a short time span. When the (NH_4_)_2_S was diluted to 1:50, the stability of the inks was notably improved, as evidenced by a sedimentation test shown in Figure S3. A planar image for the film obtained with 1:50 (NH_4_)_2_S diluted ink can be seen in Figure 8a, featuring a much more cohesive microstructure with reduced cracking and roughness. The cross-section view of the film is shown in Figure 8b, featuring a compact structure with reduced thickness and densely packed grains. Table 1 compares the elemental composition of CZTS films obtained from the dilute 1:50 (NH_4_)_2_S ink with that of the 1:9 (NH_4_)_2_S ink. The increased Sn level of the film made from the 1:50 dilution nanoink can be explained by the existence of undissociated SnS_2_, which had not been converted into the complexed [Sn_2_S_6_]^4−^ form due to the low concentration of (NH_4_)_2_S. We also expect increased Sn loss during the deposition, as excess (NH_4_)_2_S in solution may etch or complex deposited SnS_2_ when multiple spin coating layers are deposited, depleting Sn from the film prior to sulfurization [58]. Furthermore, the (NH_4_)_2_S concentration also determines the ionic strength of the solution, which, if in excess, can reduce the electric double layer surrounding nanoparticles and cause aggregation, contributing to the sedimentation of the nanoparticles in a short time span that is observed. However, the stoichiometries detected deviate from Cu-poor, Zn-rich composition found in high performance CZTS devices with more favorable defect formation [14,53], so future work will target a more favorable elemental ratio, as well as further film morphology improvement and device characterization.

## 4. Conclusions

In conclusion, a novel, nanoparticle-based process was developed for the deposition of CZTS absorber layers, making use of binary metal sulfides stabilized in a thiourea-free, aqueous nanoink with tin(IV) chalcogenide complexes. Hydrothermally prepared binary sulfide (Cu_x_S and ZnS) nanocrystals were stabilized using ammonium thiostannate(IV) complexes formed by dissolving SnS_2_ in an (NH_4_)_2_S aqueous solution, which avoids the carcinogenic thiourea reagent required in one-pot approaches. Following spin coating onto soda lime glass substrates, the thiostannate-capped binary chalcogenide nanoparticles were converted to the quaternary, crystalline kesterite CZTS phase upon sulfurization-annealing at 550 °C with minimal impurities. An optical bandgap of 1.44 eV is measured, matching theoretical calculations. Photoluminescence bands detected at room temperature indicate both band-to-band and band-to-tail recombination mechanisms, suggesting the existence of disordered kesterite in the absorber layer. The ink formulation was varied in order to improve the film morphology and microstructure. Increased nanoparticle weight fraction in the ink was beneficial for complete substrate coverage and a denser sintered film arrangement. Reducing the (NH_4_)_2_S concentration resulted in the existence of undissociated SnS_2_ nanocrystals in the ink and a higher ink stability, which benefitted the film forming properties of the ink. The present work opens the way for further improvement of the film morphology and microstructure, as well as optimizing sulfurization conditions to eliminate antisite defects that should ultimately lead to competitive CZTS photovoltaic devices from an aqueous, nanoparticle-based deposition method.

## Figures and Tables

**Table 1:** Normalized EDS composition values for constituent CZTS film elements at different dilution levels of (NH_4_)_2_S:H_2_O, standard solid concentration of 170 mg/mL.

(NH_4_)_2_S:H_2_O Dilution	Cu (at%)	Zn (at%)	Sn (at%)	S (at%)
1:9	27.2	19.4	6.4	47.0
1:50	27.8	11.8	12.4	48.0
Stoichiometric CZTS	25.0	12.5	12.5	50.0

## Supplementary Materials

The following are available online at https://www.mdpi.com/2079-4991/9/10/1382/s1, Figure S1: TEM image of hydrothermal nanocrystals of Cu_x_S and ZnS, Figure S2: XPS spectra comparison of C 1s and Cu 2p after etching adsorbed carbon contamination, Figure S3: Sedimentation test evaluating stability of the nanoink, Table S1. Lattice parameter calculations from experimental XRDs of two sulfurized CZTS thin films made with inks of different dilution ratio (NH_4_)_2_S:H_2_O and 170 mg/mL nanoparticle concentration.
