# Supplementary material for: Aqueous-Based Binary Sulfide Nanoparticle Inks for Cu2ZnSnS4 Thin Films Stabilized with Tin(IV) Chalcogenide Complexes
Source: Nanomaterials (Basel). 2019 Sep 26;9(10):1382. doi: 10.3390/nano9101382 (PMC6835281; doi:10.3390/nano9101382)
Supplement: Supplementary file 1 [file nanomaterials-09-01382-s001.pdf]

Supplementary Materials

Aqueous-based Binary Sulfide Nanoparticle Inks for Cu_2ZnSnS_4 Thin Films Stabilized with Tin(IV) Chalcogenide Complexes

Han Wang, Amrita Yasin, Nathaniel J. Quitoriano, and George P. Demopoulos *,†

Materials Engineering, McGill University, Montreal H3A 0C5, Canada; han.wang5@mail.mcgill.ca, amrita.yasin@mail.mcgill.ca, nate.quitoriano@mcgill.ca

* Correspondence: george.demopoulos@mcgill.ca

† https://orcid.org/0000-0001-8112-5339

In this section, supplementary data is provided as follows. Figure S1 is a TEM image of hydrothermal nanocrystals of Cu_xS and ZnS. The smaller nanocrystals on the order of 50 nm were shown via EDX to be ZnS, while the larger nanocrystals were found to be Cu_xS. Figure S2 provides XPS spectra after etching adsorbed carbon contamination on the surface. These spectra show that minimal carbon impurities are present within the CZTS thin films produced using our methodology. Figure S3 provides images of inks at different dilution ratios of $(NH_4)_2S$:H_2O of 1:9 (on the right) and 1:50 (on the left), using a particle concentration of 170 mg/mL. The inks were kept under ambient conditions for 2 hours with no magnetic stirring. Table S1 shows lattice parameter calculations for sulfurized CZTS thin films at different $(NH_4)_2S$:H_2O ratios. Calculations were performed using TOPAS software version 5. Khadka *et al.* (reference 41) show that the c/2a ratios of suggest the presence of the kesterite phase since they are below 1.

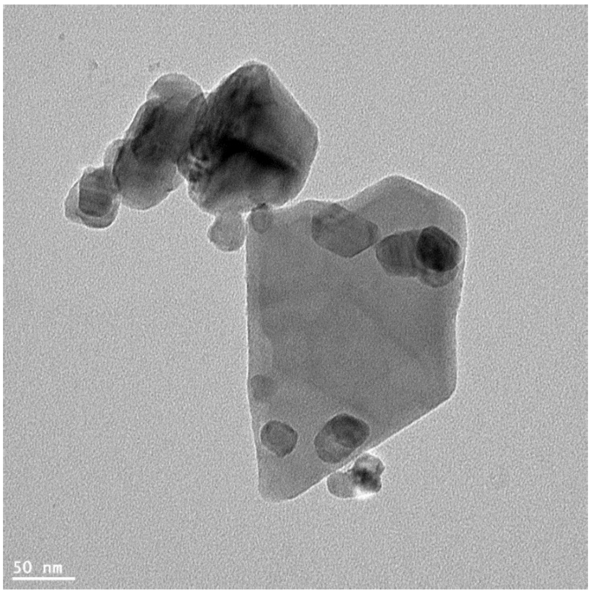

Figure S1. TEM image of hydrothermal nanocrystals of Cu_xS and ZnS.

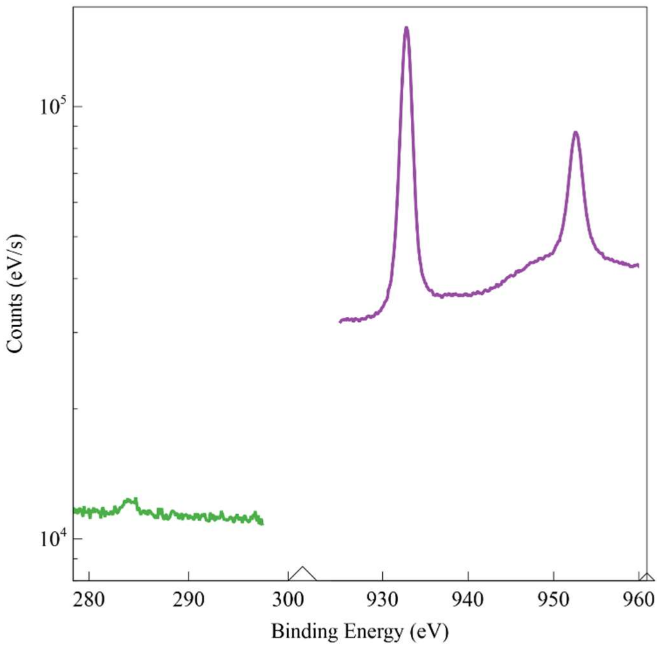

Figure S2. XPS spectra comparison of C 1s and Cu 2p after etching adsorbed carbon contamination.

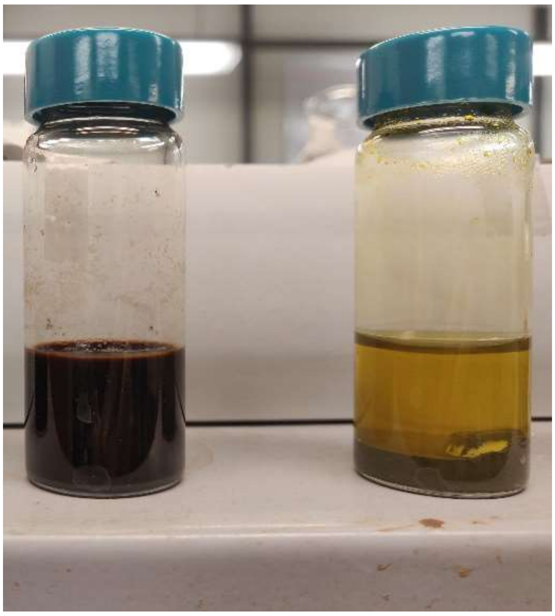

Figure S3. Sedimentation test evaluating stability of the nanoink.

Table S1. Lattice parameter calculations from experimental XRDs of two sulfurized CZTS thin films made with inks of different dilution ratio $(NH_4)_2S$:H_2O and 170 mg/mL nanoparticle concentration

$(NH_4)_2S$:H_2O Dilution	a	c	c/2a
1:9	5.4202	10.836	0.9996
1:50	5.4370	10.804	0.9936
